# *ADIPOQ* rs1063537 polymorphism correlates with diabetic kidney disease severity in southern Chinese Han patients with type 2 diabetes

**DOI:** 10.1097/MD.0000000000048028

**Published:** 2026-03-13

**Authors:** Wei Chen, Meili Lin, Huabin Wang, Miao Fu, Caiqun Huang, Shengchun Feng, Yongjun Ma

**Affiliations:** a Department of Clinical Laboratory, Affiliated Jinhua Hospital, Zhejiang University School of Medicine, Jinhua, Zhejiang, China.

**Keywords:** *ADIPOQ* gene, diabetic kidney disease, single nucleotide polymorphisms, type 2 diabetes mellitus

## Abstract

Currently, there are many studies on the relationship between the *ADIPOQ* gene polymorphisms and the onset of type 2 diabetes (T2D), and the research results vary among different regions and ethnic groups. However, there are relatively few studies on the relationship between *ADIPOQ* gene polymorphisms and the occurrence and severity of diabetic kidney disease (DKD). This study aims to investigate the association of DKD with the *ADIPOQ* gene polymorphisms rs2241766 and rs1063537 in patients with T2D in the Han population in southern China. A total of 347 patients with type 2 diabetic kidney disease (T2DKD) from the Jinhua area in central Zhejiang between 2021 and 2023 were enrolled as the case group, which was divided into the microalbuminuria phase (group I: urinary albumin-to-creatinine ratio [UACR] 30–299 mg/g, 185 cases) and the macroalbuminuria phase (group II: UACR ≥ 300 mg/g, 162 cases) based on UACR. Meanwhile, 191 patients with T2D and without kidney disease were recruited as the control group during the same period. The KASP-PCR (competitive allele-specific polymerase chain reaction) technique was used for genotyping of the rs2241766 and rs1063537 loci in the *ADIPOQ* gene, aiming to explore their associations with the occurrence and severity of T2DKD. The frequency of the TT genotype at the rs1063537 locus was 8.6% in group I and 15.4% in group II, with a statistically significant difference in distribution between the 2 groups (*P* *<* .05). After adjusting for age, gender, body mass index (BMI), diabetes duration, hypertension status, creatinine, and fasting plasma glucose, this genotype was significantly associated with the macroalbuminuria phase of T2DKD (*P* = .016), meaning that carriers of the TT genotype had a 2.47-fold higher risk of developing macroalbuminuria compared with those with the CC genotype. No statistically significant differences were observed in the genotype distributions of the rs2241766 and rs1063537 loci in the *ADIPOQ* gene between the case group and the control group (*P* > .05). In the Han population in southern China, the TT genotype at the rs1063537 locus of the *ADIPOQ* gene in patients with T2DKD is significantly associated with the presence of the macroalbuminuria stage.

## 1. Introduction

Diabetes kidney disease (DKD) is one of the most common complications of diabetes, characterized by chronic kidney disease caused by chronic hyperglycemia, involving the entire kidney (including the glomeruli, tubules, interstitium, and blood vessels).^[1]^ In China, DKD ranks as the second leading cause of end-stage renal disease. Studies have found that the onset and progression of DKD are not entirely proportional to blood sugar control and disease duration; there is a familial clustering phenomenon and ethnic differences in the onset of DKD, indicating that genetic factors play an important role in the occurrence and development of DKD.^[2]^ Genetic factors can increase susceptibility to DKD by affecting the kidney’s responsiveness to environmental factors, and their role in the occurrence of DKD should not be ignored.^[3]^ Adiponectin, also known as *ADIPOQ*, is a hormone protein secreted by adipocytes.^[4]^ The *ADIPOQ* gene, encoding adiponectin, is located on chromosome 3q27, with 2 introns and 3 exons, spanning approximately 17kb in length. Genome-wide association studies have found a relationship between chromosome 3q27 and type 2 diabetes (T2D) and DKD.^[2]^ Studies in Danish, Finnish, and French populations suggest a strong association between *ADIPOQ* polymorphisms and DKD, but these results still need further exploration and validation in the Han population in southern China.^[5]^ Therefore, this study aims to validate the previously reported associations between 2 specific *ADIPOQ* single nucleotide polymorphisms (SNPs [rs2241766 and rs1063537]) and type 2 diabetic kidney disease (T2DKD) in a Southern Han Chinese population with T2D, providing novel insights into genetic risk assessment and disease progression staging of T2DKD at the molecular level.

## 2. Methods and materials

### 2.1. Study population

A total of 347 patients with T2D and DKD admitted to the Endocrinology and Nephrology Departments at Affiliated Jinhua Hospital, Zhejiang University of Medical School, from 2021 to 2023 were collected as the case group. Among them, there were 194 males and 153 females, aged between 16.0 and 90.0 years, with a median age of 62.0 years. Concurrently, 191 patients with T2D and without kidney disease admitted to the Endocrinology Department in the same period were collected as the control group, including 106 males and 85 females, aged between 31.0 and 89.0 years, with a median age of 63.0 years. According to the “Chinese Guidelines for the Prevention and Treatment of Diabetic Kidney Disease (2021 Edition),^[6]^” the criteria for diagnosing DKD included a urinary albumin-to-creatinine ratio (UACR) ≥ 30 mg/g and/or an estimated glomerular filtration rate (eGFR) below 60 mL/min/1.73 m^2^, persisting for more than 3 months. The patients with DKD were further divided into 2 subgroups: Group I: microalbuminuria stage (UACR 30–299 mg/g)^[7]^; group II: macroalbuminuria stage (UACR ≥ 300 mg/g). The exclusion criteria for this study were: Patients with type 1 diabetes, those with nephrotic syndrome, or individuals with autoimmune kidney diseases; patients with refractory hypertension; patients with a significant increase in ACR in the short term or a rapid decline in eGFR; patients with hematuria or urinary tract infections; patients with primary renal diseases other than DKD. All study subjects were of Han ethnicity and were not blood-related to each other. This study was approved by the ethics committee of Affiliated Jinhua Hospital, Zhejiang University of Medical School (2021-IRB-211), and all patients were informed and consented to participate.

### 2.2. Data collection

Clinical data collection of the study subjects included basic information such as name, age, gender, ethnicity, and place of origin, body mass index (BMI), hypertension status, duration of diabetes, and medication use/regimen. The Beckman Coulter AU5800 automatic biochemical analyzer was used to measure creatinine (Cr), blood urea nitrogen (BUN), serum uric acid (UA), fasting plasma glucose (FPG), postprandial plasma glucose (2hPPG), total cholesterol (TC), triglycerides (TG), high-density lipoprotein cholesterol (HDL-C), and low-density lipoprotein cholesterol (LDL-C). The Bio-Rad hemoglobin analyzer D-100 was used to measure glycated hemoglobin (HbA1c) levels. The Biosystems automatic specific protein analyzer BA400 was used to measure the urinary albumin-to-creatinine ratios (UACR). Medication use included angiotensin-converting enzyme inhibitors (ACEIs) and angiotensin receptor blockers (ARBs).

### 2.3. Genetic typing

Two milliliters of peripheral blood were collected from both the case and control groups and placed into EDTA-K2 anticoagulant tubes. DNA extraction was performed following the instructions of the TIANGEN kit, and the absorbance value of the sample DNA was measured using a UV-240 ultraviolet spectrophotometer, ensuring a ratio of 1.7 to 2.0 and a DNA concentration of 30 to 50 ng/µL. The DNA samples were then stored at −80°C for future use. The AB Real-Time PCR System platform AB7900 instrument was utilized to conduct KASP-PCR (competitive allele-specific polymerase chain reaction) for the rs2241766 and rs1063537 polymorphisms of the *ADIPOQ* gene. The primer and probe sequences employed are listed in Table 1. Raw data and genetic typing charts were acquired using TYPER4.0 software (Sequenom, Inc.), and we undertook a thorough examination of data file integrity and accuracy. Subsequently, the results were saved onto the appropriate storage medium for further information analysis. Quality control was implemented by randomly selecting 5% of the samples, with a 100% consistency rate upon retesting.

**Table 1:** Primers and probe sequences at rs2241766 and rs1063537 of *ADIPOQ* gene.

Genotype	Primer sequence	Length (bp)
rs2241766		
FAM tag sequence	AGTCGTGGTTTCCTGGTCATGC	22
HEX tag sequence	GAGTCGTGGTTTCCTGGTCATGA	23
Universal sequence	GCTGGGAGCTGTTCTACTGCTATTA	25
rs1063537		
FAM tag sequence	ATTTACTCTGTTTTTGCTAAAATCTTTGAAA	31
HEX tag sequence	ATTTACTCTGTTTTTGCTAAAATCTTTGAAG	31
Universal sequence	TCAGGAGACAATAACTCCAGTGATGTT	27

### 2.4. Statistical analysis

The software SAS 9.4 (SAS Institute Inc.) was used to conduct statistical analysis on the data results. Continuous variables following a normal distribution were expressed as Mean ± SD. The difference between the means of 2 sample groups was analyzed using *t* tests. For non-normally distributed continuous variables, the median (P25, P75) was used, and group comparisons were performed using nonparametric Mann–Whitney *U* tests. Categorical variables were analyzed using the chi-square test. Goodness-of-fit tests were used to assess whether the genetic frequencies of the 2 loci in the control group conformed to Hardy–Weinberg equilibrium, with *P* *>* .05 indicating equilibrium. Univariate logistic regression analysis was employed to calculate the odds ratio (OR) and 95% confidence interval (95% CI), while multiple-factor logistic regression was utilized to assess the correlation between gene polymorphisms and T2DKD, adjusting for confounding factors. Differences in the distribution of different genotypes between case group I (microalbuminuria stage) and group II (macroalbuminuria stage) were analyzed to investigate the relationship between different genotypes and the severity of DKD. All statistical analyses were considered statistically significant at *P* < .05. In addition, we utilized SNPinfo Web Server (https://snpinfo.niehs.nih.gov/), RegulomeDB, PolymiRTS Database 3.0, and the GTEx database to perform functional prediction and expression quantitative trait locus (eQTL) analysis of candidate SNPs. The aim was to evaluate their potential impacts on transcriptional regulation, splicing regulation, miRNA binding, protein structure and function, and other related aspects.

## 3. Results

### 3.1. Comparison of clinical data between case group and control group

As shown in Table 2, patients in the T2DKD group exhibited significantly higher FPG levels compared to the type 2 diabetes mellitus (T2DM) group (8.24 ± 3.78 vs 7.03 ± 2.75 mmol/L, *P* < .01). The UA level in the T2DKD group was 341.18 ± 103.94 μmol/L, significantly higher than the 313.72 ± 85.66 μmol/L observed in the T2DM group (*P* < .01). Additionally, the median TG level in the T2DKD group was 1.63 mmol/L (interquartile range [IQR]: 1.17–2.70), significantly elevated compared to the control group’s 1.31 mmol/L (IQR: 0.92–1.94; *P* < .01). Regarding renal function parameters, the T2DKD group showed higher median Cr levels (82 μmol/L; IQR: 66–105) versus the control group (74 μmol/L; IQR: 65–83; *P* < .01). The UACR was markedly increased in the T2DKD group (median: 252.55 mg/g; IQR: 69.66–737.60) compared to controls (median: 13.38 mg/g; IQR: 4.86–20.90; *P* < .01). No significant differences were observed between groups for age, sex, 2hPPG, HbA1c, TC, HDL-C/LDL-C, or BUN levels (*P* > .05). However, BMI, hypertension status, diabetes duration, and ACEI/ARB usage show significant intergroup differences (*P* < .05).

**Table 2:** Comparison of clinical data between T2DKD group and T2DM group.

Demographic characteristics	T2DM (n = 191)	T2DKD (n = 347)	Statistical value	*P* value
Age (*x̅ *± *s*, yr)	61.20 ± 11.19	60.73 ± 13.26	*t *= 0.44	.66
Gender (male/female, n)	106/85	194/153	*χ*^2^* *= 0.01	.93
BMI (kg/m^2^)	23.95 ± 2.87	25.36 ± 3.60	*t *= 4.67	<.01
Hypertension (n, %)	98 (51.31)	268 (77.23)	*χ*^2^* *= 38.07	<.01
Diabetes duration (yr)	7 (2, 12)	10 (5, 17)	*Z *= 5.42	<.01
ACEI/ARB use (n, %)	47 (24.61)	134 (38.62)	*χ*^2^* *= 10.83	<.01
FPG (*x̅ *± *s*, mmol/L)	7.03 ± 2.75	8.24 ± 3.78	*t *= 4.25	<.01
2hPPG (*x̅ *± *s*, mmol/L)	10.74 ± 3.93	11.37 ± 4.71	*t *= 1.65	.10
HbAlc (%)	8.59 ± 2.37	8.85 ± 2.27	*t *= 1.24	.22
TC (*x̅ *± *s*, mmol/L)	4.31 ± 1.34	4.50 ± 1.39	*t *= 1.52	.13
UA (*x̅ *± *s*, mmol/L)	313.72 ± 85.66	341.18 ± 103.94	*t = *3.29	<.01
TG, *M (P*_25_, *P*_75_), mmol/L	1.31 (0.92, 1.94)	1.63 (1.17, 2.70)	*Z *= 4.59	<.01
HDL-C (*x̅* ± *s*, mmol/L)	1.12 ± 0.44	1.09 ± 0.31	*t *= 0.90	.37
LDL-C (*x̅* ± *s*, mmol/L)	2.69 ± 0.87	2.84 ± 0.94	*t *= 1.89	.06
Cr, *M (P*_25_, *P*_75_), μmol/L	74 (65, 83)	82 (66, 105)	*Z *= 4.18	<.01
BUN, *M (P*_25_, *P*_75_), mmol/L	5.32 (4.58, 6.76)	6.53 (5.28, 8.59)	*Z *= 6.17	<.01
UACR, *M (P*_25_, *P*_75_), mg/g	13.38 (4.86, 20.9)	252.55 (69.66, 737.6)	*Z *= 19.21	<0.01

2 hPPG = 2h postprandial glucose, ACEI = angiotensin-converting enzyme inhibitors, ARB = angiotensin receptor blockers, BMI = body mass index, BUN = blood urea nitrogen, Cr = creatinine, FPG = fasting plasma glucose, HbAlc = glycosylated hemoglobin, HDL-C = high-density lipoprotein cholesterol, LDL-C = low-density lipoprotein cholesterol, TC = total cholesterol, TG = triglyceride, T2DKD = type 2 diabetic kidney disease, T2DM = type 2 diabetes mellitus, UA = uric acid, UACR = urine albumin/creatinine ratio.

### 3.2. Multivariate logistic regression analysis of nongenetic factors for DKD

**Figure 1.**
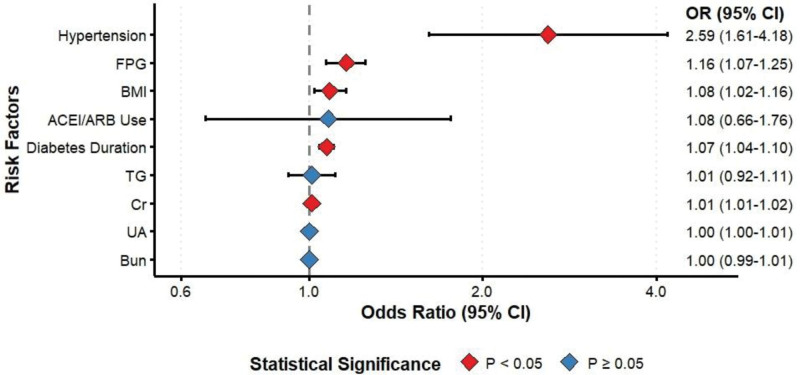
Forest plot of factors influencing T2DKD. This was a multivariate Logistic regression analysis that included statistically significant indicators from univariate comparisons as independent variables and T2DKD as the dependent variable. ACEI = angiotensin-converting enzyme inhibitor, ARB = angiotensin receptor blocker, BMI = body mass index, BUN = blood urea nitrogen, CI = confidence intervals, Cr = creatinine, FPG = fasting plasma glucose, OR = odds ratio, T2DKD = type 2 diabetic kidney disease, TG = triglyceride, UA = uric acid.

Using the 9 statistically significant variables (BMI, hypertension status, diabetes duration, ACEI/ARB use, FPG, UA, TG, Cr, and BUN) that differed between the T2DKD and T2DM groups in Table 2 as independent variables, with T2DKD as the dependent variable, we performed multivariate logistic regression analysis. As shown in Figure 1, FPG emerged as an independent risk factor for T2DKD (OR = 1.16, 95% CI: 1.07–1.25, *P* < .05). Cr levels also showed significant association with T2DKD risk (OR = 1.01, 95% CI: 1.01–1.02, *P* < .05). Additionally, BMI (OR = 1.09, 95% CI: 1.02–1.16), history of hypertension (OR = 2.59, 95% CI: 1.61–4.18), and diabetes duration (OR = 1.07, 95% CI: 1.04–1.10) were all identified as independent risk factors for T2DKD (*P* < .05).

### 3.3. Association analysis between ADIPOQ gene polymorphism and population susceptibility of DKD

The genotype distributions of *ADIPOQ* gene rs1063537 and rs2241766 in the T2DM control group both conformed to Hardy–Weinberg equilibrium (HWE test *P*-values: .183 and .641, respectively). After adjusting for age, sex, BMI, diabetes duration, hypertension, Cr and FPG, neither SNP showed significant differences in genotype distribution between the T2DKD group and T2DM controls. For rs1063537, the TT genotype frequency was 11.8% (41/347) in the T2DKD group, comparable to 9.9% (19/191) in controls (adjusted OR = 1.47, 95% CI: 0.75–2.90, *P* = .27). Similarly, the GG genotype frequency of rs2241766 was 8.9% (31/347) in T2DKD patients versus 8.4% (16/191) in controls (adjusted OR = 1.27, 95% CI: 0.60–2.67, *P* = .54), as detailed in Table 3.

**Table 3:** Association analysis between *ADIPOQ* gene polymorphism and T2DKD population susceptibility.

Genotype	T2DKD, n (%)	T2DM, n (%)	Crude OR (95% CI)	*P*	Adjusted OR (95% CI)	*P* *
rs1063537						
CC	185 (53.3)	102 (53.4)	1.00 (Ref)	–	1.00 (Ref)	–
CT	121 (34.9)	70 (36.7)	0.95 (0.65–1.40)	.805	1.22 (0.79–1.88)	.38
TT	41 (11.8)	19 (9.9)	1.19 (0.66–2.16)	.567	1.47 (0.75–2.90)	.27
rs2241766						
TT	186 (53.6)	101 (52.9)	1.00 (Ref)	–	1.00 (Ref)	–
TG	130 (37.5)	74 (38.7)	0.95 (0.66–1.39)	.805	1.24 (0.81–1.9)	.32
GG	31 (8.9)	16 (8.4)	1.05 (0.55–2.02)	.878	1.27 (0.6–2.67)	.54

BMI = body mass index, CI = confidence intervals, OR = odds ratio, T2DKD = type 2 diabetic kidney disease, T2DM = type 2 diabetes mellitus.

* Represents the *P*-value after adjustment for confounding variables (age, sex, BMI, diabetes duration, hypertension status, creatinine, and fasting plasma glucose).

### 3.4. *Association analysis of the polymorphism at 2 sites of the* ADIPOQ *gene with the severity of DKD*

**Figure 2.**
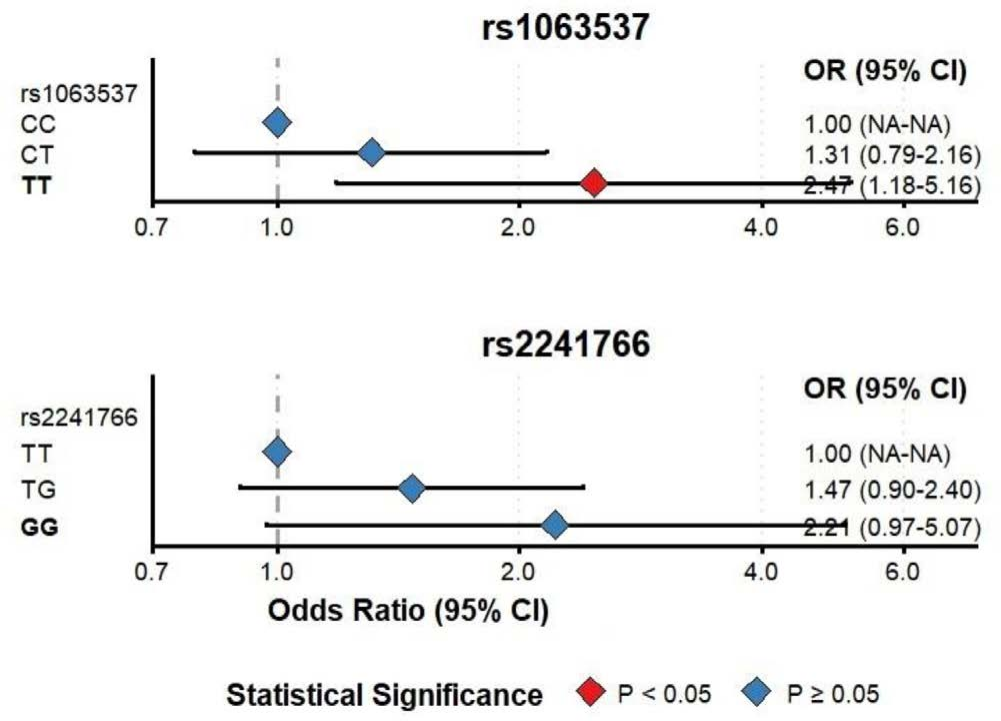
Forest plot showing the association between genotypes of rs1063537 and rs2241766 and the severity of type 2 diabetic kidney disease (T2DKD). Logistic regression analysis was adjusted for age, gender, BMI, duration of diabetes, hypertension status, Cr and fasting blood glucose. BMI = body mass index, CI = confidence intervals, Cr = creatinine, NA = not available, OR = odds ratio, T2DKD = type 2 diabetic kidney disease, TG = triglycerides.

The TT genotype frequency at rs1063537 was significantly higher in the macroalbuminuria stage (group II: 15.4% [25/162]) compared to the microalbuminuria stage (group I: 8.6% [16/185]; *P < *.05). After adjusting for confounding factors, TT genotype carriers showed a 2.47-fold increased risk of developing macroalbuminuria versus CC genotype carriers (adjusted OR = 2.47, 95% CI: 1.18–5.16, *P* = .016). Although this difference reached nominal statistical significance (*P* < .05), the relatively small sample size in the TT genotype subgroup warrants cautious interpretation. In contrast, rs2241766 genotype distributions did not differ significantly across disease severity groups. The GG genotype frequency was 11.1% (18/162) in group II versus 7.0% (13/185) in group I, with an adjusted OR of 2.21 (95% CI: 0.97–5.07) that did not reach statistical significance (*P* = .06), as detailed in Table 4 and Figure 2.

**Table 4:** Correlation analysis between *ADIPOQ* gene polymorphism and T2DKD disease severity.

Genotype	T2DKD I group, n (%)	T2DKD II group, n(%)	Crude OR (95% CI)	*P*	Adjusted OR (95% CI)	*P* *
rs1063537						
CC	105 (56.8)	80 (49.4)	1.00 (Ref)	–	1.00 (Ref)	–
CT	64 (34.6)	57 (35.2)	1.17 (0.74–1.85)	.506	1.31 (0.79–2.16)	.296
TT	16 (8.6)	25 (15.4)	**2.05 (1.03–4.10**)	**.042**	**2.47 (1.18–5.16**)	**.016**
rs2241766						
TT	106 (57.3)	80 (49.4)	1.00 (Ref)		1.00 (Ref)	
TG	66 (35.7)	64 (39.5)	1.29 (0.82–2.02)	.275	1.47 (0.90–2.40)	.122
GG	13 (7.0)	18 (11.1)	1.84 (0.85–3.96)	.123	2.21 (0.97–5.07)	.06

I group: UACR value 30.0–299.9 mg/g; II group: UACR value ≥300.0 mg/g.

Bold values indicate statistically significant differences compared to the control group (*P* < .05).

BMI = body mass index, CI = confidence intervals, OR = odds ratio, T2DKD = type 2 diabetic kidney disease, UACR = urinary albumin-to-creatinine ratio.

* Represents the *P*-value after adjustment for confounding variables (age, sex, BMI, diabetes duration, hypertension status, creatinine, and fasting plasma glucose).

#### 3.4.1. Functional prediction analysis of 2 SNPs

Bioinformatics analysis revealed that rs1063537 is localized in the 3′-untranslated region (3′-UTR) of the *ADIPOQ* gene (chr3:188056769). Predicted as a miRNA-binding site by SNPinfo Web Server, allelic variations at this locus may affect the binding efficiency between miRNAs and target mRNAs. No significant functional impacts were detected at transcription factor binding sites (TFBS), splice sites, or nonsynonymous single nucleotide polymorphism (nsSNP) regions, indicating that it exerts biological effects primarily through posttranscriptional regulation. In terms of population genetic characteristics, rs1063537 is a common polymorphism in Asian populations (C allele frequency approximately 70–75%) with stable population distribution. Annotation from PolymiRTS Database 3.0 classified its T allele as Class D, suggesting that this derived allele may disrupt evolutionarily conserved miRNA-binding sites. This disruption could impede the targeted regulation of *ADIPOQ* mRNA by miRNAs, thereby modulating phenotypes through mechanisms such as altering mRNA stability, influencing translation efficiency, or disrupting the balance of gene regulatory networks. The evolutionarily conserved nature of this locus also implies its significant functional importance. Rs2241766 is situated within the *ADIPOQ* gene (chr3:188053586). It was predicted to affect splice regulatory domains (abolish domain), which may further alter mRNA splicing patterns and the expression of protein isoforms. However, this locus exhibits an extremely low conservation score (conservation = 0.000), indicating lack of evolutionary conservation. It may represent a recently emerged functional locus or a neutral variant. No significant functional impacts were detected in TFBS, miRNA binding, nsSNP, or stop codon regions.

## 4. Discussion

Diabetic kidney disease is a prevalent microvascular complication in patients with T2D, which has the potential to progress to end-stage kidney disease. Research indicates that approximately one-third of diabetes patients globally will ultimately develop DKD, necessitating treatments such as hemodialysis or kidney transplantation, leading to significant patient distress and imposing substantial financial burdens.^[8]^ Therefore, investigating the risk factors associated with the onset and progression of DKD is of paramount importance.^[9]^ The precise pathogenesis of DKD remains unclear, with the consensus being that it is a multifaceted condition influenced by genetic and environmental factors. There exists genetic and phenotypic heterogeneity among different races, ethnicities, and geographical regions in the context of DKD. In this study, clinical data from 347 individuals with T2D and DKD (case group) were compared with data from 191 individuals with T2D but no kidney disease (control group). The analysis revealed significantly higher levels of FPG, UA, TG, Bun, and Cr in the case group compared to the control group (*P* < .05). Subsequent multivariate logistic regression analysis confirmed that FPG and Cr are nongenetic independent risk factors associated with the incidence of DKD (*P* *<* .05). These results were similar to previous research findings.^[10,11]^

Genetic factors can increase the susceptibility to DKD by regulating the kidney’s responsiveness to environmental factors and play a crucial role in the occurrence and progression of the disease. As a bioactive factor secreted by adipocytes, adiponectin participates in the pathophysiological processes of diabetes, metabolic syndrome, and other diseases through anti-inflammatory, anti-fibrotic, and antioxidant mechanisms.^[5]^ Additionally, serum adiponectin levels are negatively correlated with T2D, insulin resistance, and metabolic syndrome.^[12-14]^ *ADIPOQ* is the coding gene for adiponectin, and the role of its polymorphisms in type 2 diabetes-related kidney disease (T2DKD) remains unclear. Studies have suggested that *ADIPOQ* gene variants are associated with renal risk in subjects with T2D and early-stage renal dysfunction.^[15]^ This study found no significant association between the rs2241766 and rs1063537 loci of the *ADIPOQ* gene and the risk of T2DKD, which is inconsistent with some previous studies: a French prospective study suggested that the G allele of rs2241766 is a risk factor for T2DKD^[14]^; a meta-analysis of Caucasian and African populations indicated that the GG genotype of *ADIPOQ* rs2241766 may increase the risk of DKD^[16]^; while a Taiwanese study reported that the TT genotype of rs2241766 or the CC genotype of rs1063537 is associated with an elevated risk of T2DKD in local male patients with T2D.^[17]^ This inconsistency may be related to 3 factors: first, differences in the linkage disequilibrium structure of the *ADIPOQ* gene region among different populations lead to distinct association patterns between target single nucleotide polymorphisms (SNPs) and pathogenic variants; second, the interaction between environmental factors (such as diet and lifestyle) and genetic background may modify genetic effects; third, this study focuses on the Han population in southern China, whereas previous studies included populations from Europe, Africa,^[18]^ and Taiwan. These findings suggest significant population heterogeneity in the genetic architecture of DKD, and these 2 SNPs may not be the main genetic determinants of T2DKD susceptibility in the Han population in southern China.

A study in the Korean population suggested that the GG genotype of the *ADIPOQ* gene rs2241766 may be associated with elevated urinary albumin-to-creatinine ratio (UACR) and an increased risk of DKD.^[19]^ The median time to DKD onset in carriers of this genotype was 2 years shorter than that in those with other genotypes, but the difference did not reach statistical significance. In the present study, analysis of the genotype distribution of *ADIPOQ* gene rs2241766 and rs1063537 loci in patients with T2DKD in the microalbuminuria phase (group I) and macroalbuminuria phase (group II) revealed no significant difference in the GG genotype of rs2241766 between the 2 groups (OR = 2.21, 95% CI: 0.97–5.07, *P* = .06). In contrast, the distribution frequency of the TT genotype of rs1063537 in group II was significantly higher than that in group I (adjusted OR = 2.47, 95% CI: 1.18–5.16, *P* < .05), suggesting that this genotype may be associated with the severity of T2DKD. It should be noted that the subgroup sample size of the rs1063537 TT genotype was small (16 cases in group I and 25 cases in group II), resulting in limited statistical power. Although the association was statistically significant in the present cohort, the effect size is a preliminary result whose stability requires further verification. Larger-scale prospective studies are needed to confirm its genetic effect in subgroup analysis. Additionally, this study adopted a cross-sectional design, only reflecting the association at a specific time point. Although the rs1063537 TT genotype was more prevalent in patients with macroalbuminuria, it cannot be confirmed that it accelerates DKD progression. It is hypothesized that this genotype is more likely a marker of the highly aggressive disease phenotype rather than a direct driver of disease progression. Subsequent longitudinal cohort studies starting with patients in the microalbuminuria phase are required to clarify its causal role in DKD progression.

Although rs1063537 is located in the 3′-untranslated region (3′-UTR) of the *ADIPOQ* gene, our bioinformatics functional prediction analysis provides clues to its biological significance. Expression quantitative trait locus (eQTL) analysis of this locus in tissues via the GTEx database revealed no significant effect of rs1063537 on the differential expression of *ADIPOQ*. However, this locus is a miRNA-binding site, and the region where it resides is typically involved in the regulation of mRNA stability, localization, and translation efficiency. Therefore, we hypothesize that rs1063537 may alter the stability or translation process of *ADIPOQ* mRNA, thereby affecting the level or biological activity of circulating adiponectin (especially the high-molecular-weight [HMW] isoform) and consequently influencing the severity of DKD. This hypothesis awaits validation through functional experiments in cellular or animal models in the future.

## 5. Study limitations

Although this study clearly demonstrates a significant association between the TT genotype of the *ADIPOQ* gene rs1063537 and the severity of T2DKD, it still has the following limitations: lack of mechanistic validation data – without detection data on serum adiponectin levels and their subtype distribution, as well as failure to conduct functional experiments, makes it impossible to construct a causal pathway among *ADIPOQ* gene variation, adiponectin expression, and the severity of the observed T2DKD phenotype; certain *ADIPOQ* gene polymorphisms (e.g., rs2241766) have been demonstrated to regulate serum adiponectin concentrations via a dose–response effect,^[19,20]^ while adiponectin can influence renal function through mechanisms such as improving insulin sensitivity, exerting anti-inflammatory effects, and inhibiting atherosclerosis,^[21,22]^ while the lack of relevant biochemical data and functional validation in this study restricts the accurate interpretation of the biological effects of the rs1063537 polymorphism; limited generalizability of results – the study samples are focused on the Southern Han population in Jinhua, Zhejiang Province, and the specificity of genetic backgrounds and environmental factors makes it difficult to extrapolate the conclusions to populations of other ethnicities or geographical regions, requiring multicenter, cross-population studies to verify their universality; insufficient statistical power – the relatively small sample size of the rs1063537 TT genotype subgroup reduces the robustness and precision of effect size estimation, which may increase the risk of false positive results or overestimation of effects; limitations in causal inference – the cross-sectional study design can only reveal the association between genotype and disease severity, but cannot clarify the temporal sequence of disease progression from microalbuminuria to macroalbuminuria, making it difficult to establish a causal relationship between the 2; narrow scope of genetic analysis – only 2 candidate loci (rs2241766 and rs1063537) within the *ADIPOQ* gene were analyzed, without covering other variations, haplotype structures, or linkage disequilibrium patterns of the gene, thus failing to exclude the potential impact of other regions within the gene or variation combinations on T2DKD susceptibility. These existing limitations suggest that future studies should integrate longitudinal cohort designs, measurements of serum adiponectin levels, and expand the range of loci for molecular functional experiments and association analyses in multicenter and cross-population settings, so as to elucidate the potential biological pathways through which this genetic variant influences DKD phenotypes.

## 6. Conclusions

In this Southern Han Chinese cohort with T2D, polymorphisms of the *ADIPOQ* gene at rs2241766 and rs1063537 may not represent risk factors for the development of T2DKD. This observation helps explain the inconsistency with findings from some other populations and underscores the population-specific nature of the disease’s genetic background. However, the TT genotype of rs1063537 is associated with an increased risk of developing macroalbuminuria in patients with T2DKD. This finding suggests that the role of this locus may be stage-specific, and this associative discovery provides a clue for subsequent validation of the locus’s function in larger sample sizes and diverse populations, as well as for conducting in-depth functional mechanism studies – with particular attention to its impact on disease progression rather than mere disease onset.

## Acknowledgments

We thank all the participants and all the colleagues of department of Clinical Laboratory, Affiliated Jinhua Hospital, Zhejiang University School of Medicine. WC and ML are the guarantors of this work and, as such, have full access to all the data in the study and take responsibility for the integrity of the data and the accuracy of the data analysis.

## Author contributions

**Data curation:** Meili Lin, Shengchun Feng.

**Methodology:** Huabin Wang, Caiqun Huang.

**Software:** Miao Fu.

**Writing – original draft:** Wei Chen.

**Writing – review & editing:** Yongjun Ma.
